# Editorial: Women in Lanthanide-Based Luminescence Research: From Basic Research to Applications

**DOI:** 10.3389/fchem.2021.667672

**Published:** 2021-03-23

**Authors:** Qianqian Su, Lining Sun, Eva Hemmer, Ho Seong Jang

**Affiliations:** ^1^ Institute of Nanochemistry and Nanobiology, Shanghai University, Shanghai, China; ^2^ Research Center of Nano Science and Technology, College of Sciences, Shanghai University, Shanghai, China; ^3^ Department of Chemistry and Biomolecular Sciences, University of Ottawa, Ottawa, ON, Canada; ^4^ Materials Architecturing Research Center, Korea Institute of Science and Technology, Seoul, South Korea

**Keywords:** lanthanide, luminescence, upconversion (UC) luminescence, downshifting, nanomaterials

**Editorial on the Research Topic** **Women in Lanthanide-Based Luminescence Research: From Basic Research to Applications**Lanthanide-based luminescence nanomaterials have attracted considerable attention in the last 2 decades because of the unique optical properties of lanthanides. Given their 4f electronic configuration, these nanomaterials exhibit unique emission spectra, including tunable emissions spanning the ultraviolet (UV) to near-infrared (NIR) regions, long excited-state lifetime, high resistance to optical photobleaching, no optical blinking, superior photostability, as well as large Stoke or anti-Stoke shifts. This set of outstanding features makes the study of lanthanide-based luminescence nanomaterials a subject of fundamental and technological importance (Auzel, 2004; Wang et al., 2010; Bettinelli et al., 2015; Li et al., 2015; Jalani et al., 2018). Indeed, encouraged by recent advances in the controllable synthesis and optical tuning of lanthanide-based nanomaterials, there are high expectations for the lanthanide research field to shape a wider attention on the emerging applications, including bioimaging, multiplexing sensing, super-resolution imaging, opto-genetics, volumetric display, photovoltaics, and more (Chen et al., 2014; Chen et al., 2015; Zhou et al., 2015; Liu et al., 2017; Su et al., 2017; Chen et al., 2018; Gai et al., 2018; Ma et al., 2019).

Among, many inspiring female researchers have made great contributions to the lanthanide research field. These contributions drove forward the understanding of the underlying mechanisms of lanthanide luminescence and pushed the exploration of manifold possible applications. Moreover, the spirit of the recent “Research Topic on Women in Science: Chemistry” motivated us to initiate a research topic focusing on the contributions of female researchers in lanthanide research. We, the editors of this special issue hope to help with “Women in Lanthanide-based Luminescence Research: From Basic Research to Applications” to motivate closure of the gender gap in this field, which—despite all stories of success—is still present. As such, the aim of this research topic is to promote the excellent female researchers’ work in theory, experimental methodology, as well as emerging applications of lanthanide-based nanomaterials.

A key challenge in lanthanide-based upconversion nanoparticles is the low quantum efficiency of the upconversion process. While promising proof-of-concepts have demonstrated the great application potential for lanthanide-based nanomaterials in e.g., photodynamic therapy, light-triggered drug release, photocatalysis or (solar) energy conversion technologies, the success of these approaches in real life depends on the community’s capability to overcome the limitations of low upconversion quantum efficiency. Consequently, great efforts are undertaken by researchers around the globe to tackle this challenge.

For example, Pei et al. surveyed examples of recent optical enhancement strategies on upconversion and even downshifting luminescence by metal ions doping strategy. Special attention is paid to the related mechanism of enhancement, involving host lattice asymmetry increase and energy transfer pathway optimization. Another strategy to boost upconversion efficiency is broadband dye-sensitization to partially overcome the limitation of the small absorption cross-sections and fixed excitation wavelength of the lanthanides. Agbo et al. reported the ultraviolet ligand decorated core-shell lanthanide nanoparticles, resulting in a large spectral shift, shifting the excitation wavelength to the UV range and resulting in the enhancement of NIR emission.

With respect to optical and biomedical applications, the assemblies of luminescence nanoparticles with unique structures hold great promise. Herein, merging several materials classes has the power to leverage the desirable features of each moiety into one hybrid system of superior performance. However, alignment of isotropic nanoparticles into anisotropic superstructures is a fundamental challenge in nanochemistry. Hence, the controllable assembly of isotropic nanocrystals into anisotropic structures by mediating inter-particle interactions as demonstrated by Su et al. constitutes an excellent example for the successful integration of lanthanide-based nanoparticles into a polymeric structure. In their contribution, the authors developed an amphiphilic block copolymer coating approach for the preparation of the belt-like assemblies of upconversion nanoparticles with length up to micro-scale.

With features of environment-sensitive, selective and bio-compatible spectroscopic characteristics, lanthanide-doped nanoparticles are ideal for optical trapping. So-called optical tweezers are of great promise for applications ranging from single-particle spectroscopy and tailored particle assembly to high-resolution surface studies, surface-enhanced spectroscopy, and controlled investigation of biological processes. In their contribution, Ortiz-Rivero et al. highlighted recent strategies to increase the optical forces in the manipulation of lanthanide-doped nanoparticles, for example, surface modification of these nanoparticles and optimization of the optical tweezer setup to ease access of lanthanide-based nanoparticles to these exciting applications.

The environment-sensitive spectroscopic characteristics of the lanthanides further render them ideal candidates for use in chemical and biological sensing. In this context, the lanthanide nanoparticles should exhibit good water dispersibility. However, most lanthanide-doped nanocrystals are prepared by synthetic methods that involve the use of capping hydrophobic, long alkyl chain ligands. Consequently, controlled and application-oriented surface modifications are essential to improve the sensitivity and versatility of upconversion probes. Burgess et al. reported a robust and versatile method for the covalent attachment of a wide variety of molecules including organic dyes, enzymes and proteins to the surface of upconversion nanophosphors. Su et al. focused on the detection of Cu^2+^ by using water-dispersible poly(acrylic acid) (PAA)-coated upconversion nanoparticles. Wang et al. utilized the ultra-weak chemiluminescence of lanthanide nanoparticles to demonstrate the potential use in nitrite detection.

In the context of biomedicine, the use of NIR light as an excitation source, but also the emission of NIR photons, is particularly appealing. Encouraged by the deeper penetration depth, minimized background fluorescence interference and less photo-damage to normal tissues offered by NIR-excited and -emitting lanthanide-based nanoparticles, researchers have paid considerable attention to their biomedical applications. For instance, Chen et al. explored recent advances in the development of photoimmuno-therapeutic agents activated by multifunctional upconversion nanoparticles. The fascinating long lifetime of lanthanides can create a temporal coding dimension for biomedical applications. In another excellent contribution, Qin et al. illustrated the aspects of synthesis of lanthanide-doped persistent luminescence phosphors with NIR emissions and exemplify the potential impact of this research area on biomedical science, including biosensing, bioimaging, drug delivery, as well as phototherapy.

We deeply appreciate our contributors for their dedication and the reviewers for their constructive comments and suggestions. We would like to thank all the editorial staff of Frontiers in Chemistry for valuable assistance in organizing this issue. Last but not least, we hope that this themed issue will not only provide a timely overview of current developments in the field of lanthanide-based luminescence nanomaterials, offering an enjoyable and enlightening lecture to our readership, but will also encourage the career developments of female researchers in this exciting field of research.
